# Paternity Acknowledgment in 2 Million Birth Records from Michigan

**DOI:** 10.1371/journal.pone.0070042

**Published:** 2013-07-22

**Authors:** Douglas Almond, Maya Rossin-Slater

**Affiliations:** 1 Department of Economics and School of International and Public Affairs, Columbia University, New York, New York, United States of America; 2 Department of Economics, University of California Santa Barbara, Santa Barbara, California, United States of America; University of California, San Francisco, United States of America

## Abstract

Out-of-wedlock childbearing is more common in the U.S. than in other countries and becoming more so. A growing share of such non-marital births identify the father, which can create a legal entitlement to child support. Relatively little is known about individual determinants of the decision to establish paternity, in part because of data limitations. In this paper, we evaluate all birth records in Michigan from 1993 to 2006, which have been merged to the paternity registry. In 2006, 30,231 Michigan children, almost one quarter of all Michigan births, were born to unmarried mothers and had paternity acknowledged. We find that births with paternity acknowledged have worse outcomes along various health and socio-economic dimensions relative to births to married parents, but better outcomes relative to births to unmarried parents without paternity acknowledgement. Furthermore, unmarried men who father sons are significantly more likely to acknowledge paternity than fathers of daughters.

## Introduction

The non-marital fertility rate has been rising in the United States over the last several decades. In 2009, 41 percent of all births were out-of-wedlock. [1] Since single-mother households are economically disadvantaged on average – in 2010, 43 percent of children in single-mother households lived below the poverty line [2] – policymakers have been increasingly concerned with implementing measures to promote greater family involvement and financial support from non-resident fathers. Paternity acknowledgement can serve as a crucial first step in the process of securing support from unmarried fathers since it is a requirement for establishing a legal child support order. Currently, paternity acknowledgement, which is a legal procedure that is only applicable to fathers who are not married to their children's mothers, usually occurs at the time of the child's birth at the hospital. In most cases, both parents are required to be present at the hospital, and must fill out and sign a form to acknowledge paternity. Prior evidence from the Fragile Families and Child Well-Being Study suggests that among children born out-of-wedlock, paternity acknowledgement is associated with increased formal and informal child support payments and father-child visitation. [3] Child support and father-child contact are in turn positively associated with child mental health and well-being, [4] suggesting the potential role of paternity acknowledgement in child development and social welfare policy.

The existing literature on paternity acknowledgement is generally limited to studies examining impacts of in-hospital voluntary paternity establishment programs,[3], [5]–[9] which now operate nationwide. Yet analysis of the individual determinants of paternity acknowledgement is also informative. For example, understanding which factors are associated with voluntary paternity acknowledgement at birth may aid in the development of programs aimed at families who are most “at-risk” of failing to establish paternity. A few studies have conducted such analyses using survey data with relatively small sample sizes. [3], [10] We use a novel data set consisting of the universe of individual birth records in Michigan over 1993–2006 to document the trend in paternity acknowledgement over time and to conduct a comprehensive analysis of the maternal and child-specific factors associated with paternity acknowledgement at birth. Additionally, we pay special attention to the relationship between child sex and paternity acknowledgement because: i) previous findings suggest child sex may affect the marriage decision [11], and; ii) child sex is a relatively stochastic variable that might allow for a more causal interpretation of the effect on paternity.

We document a substantial increase in paternity acknowledgement rates over time in Michigan: fewer than one-tenth of all births had paternity acknowledged in 1993, while nearly one-quarter had paternity acknowledged by 2006. Over the same time period, the rate of non-marital births without paternity acknowledgement fell correspondingly from over one-quarter of all births to less than 15 percent. We also find evidence of a socio-economic status gradient in parental relationship status: in terms of health and demographic characteristics, births with paternity acknowledgement are situated in the middle between births to unmarried parents without paternity acknowledgement and births to married parents. Finally, we show interesting patterns between child gender and parental relationship status. Among unmarried births, male children are more likely to have paternity acknowledged than female children. However, at least in Michigan, the relationship between child gender and marriage is less robust than what has been previously documented in other work. [11].

## Methods

### Study Design and Population

We conducted a population-based cohort study using data from individual vital statistics natality records covering all live births in Michigan from January 1, 1993 to December 31, 2006. These data contain a categorical variable with three mutually exclusive categories: one parent on the birth certificate, two parents on the birth certificate, and acknowledgement of paternity. We infer that records with one parent on the birth certificate refer to children borne by unmarried mothers, while records with both parents on the birth certificate most likely refer to children born within married households.

In addition to information on named parents and paternity acknowledgement, we extracted the following variables from the birth records data: child's date of birth, child's gender, birth weight (in kilograms), presence of any abnormal conditions or congenital anomalies, mother's age, mother's education, mother's race and ancestry, and the number of previous live births. Our initial sample of analysis consisted of 1,859,858 births. In regression analysis, our sample was further limited to observations with non-missing data on the outcome and covariates and consisted of 1,859,473 births. Note that in our data, there was only one observation with missing data on parental relationship status. The remaining 384 births were omitted due to missing data on covariates.

### Statistical Analysis

We estimated multinomial logit models using STATA (release 11) statistical software with a categorical outcome that takes on three mutually exclusive values: one parent on the birth certificate, two parents on the birth certificate, and acknowledgement of paternity. We included the following covariates: an indicator for male child, an indicator for the birth occurring on a weekend, an indicator for any abnormal conditions or congenital anomalies, birth weight in kilograms, indicators for mother's age (20–24 years, 25–34 years, 35–44 years, 45 or more years; less than 20 years omitted), indicators for mother's education (high school degree, some college, college or more, missing education; less than high school omitted), indicators for mother's race and ethnicity (black, American Indian, Hispanic, other/unknown race; white omitted), indicators for birth parity (second, third, fourth or higher; first omitted), and the birth year. Weekend birth was analyzed because of previous findings that hospital staffing is reduced on weekends (e.g. [12]), which could potentially include staff responsible for paternity registration. We did not include any information on the fathers as covariates because presence of information about the father is very highly correlated with paternity acknowledgement status. Consequently, almost all birth records without paternity acknowledgement have missing data for father's age, father's education, and father's race/ethnicty. For example, out of 306,214 records with one parent on the birth certificate and without paternity acknowledgement, only 533 have non-missing data for father's age. Standard errors were adjusted for heteroskedasticity using the “Robust” STATA command.

## Results

**Figure 1:**
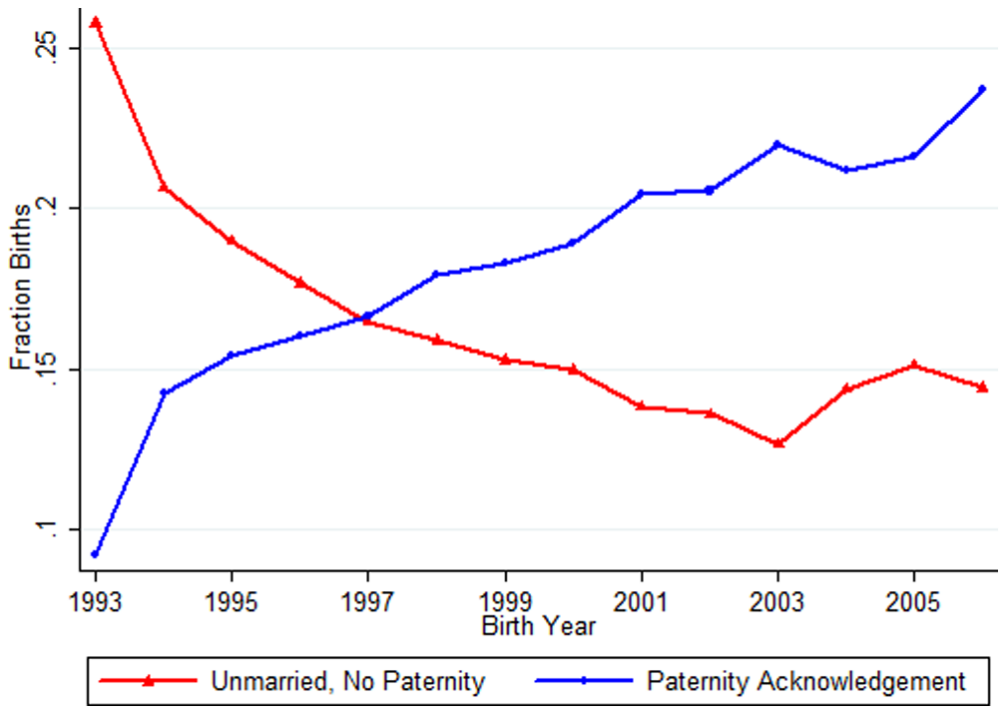
Parental Relationships Over Time, Michigan, 1993–2006. Notes: The sample is the universe of birth records in Michigan with non-missing information on “named parents on the birth certificate” over 1993–2006.

Table 1 shows the trends in the number of births, the number of unmarried births, and the number of unmarried births with paternity acknowledged over 1993–2006 in Michigan, while Figure 1 presents the trends in parental relationships over this time period in graphical form. The increase in the paternity acknowledgement rate is striking: the fraction of births with paternity acknowledged rises from less than 10 percent to nearly 25 percent over this time period. Much of this increase comes from unmarried parents being more likely to establish paternity – the rate of births with only one parent named on the birth certificate and with no paternity acknowledgement falls from over 25 percent to around 15 percent. However, the relationship is not a perfect inverse, suggesting that at least some of the change is also coming from a decrease in marriage (or, two parents being named on the birth certificate).

**Table 1:** Births Over Time, Michigan 1993–2006.

Year	Total Births	Unmarried Births	Unmarried Births with Paternity Acknowledged
1993	139,560	48,848	12,853
1994	137,844	48,107	19,634
1995	134,169	46,150	20,662
1996	133,231	44,836	21,316
1997	133,549	44,191	22,224
1998	133,649	45,191	23,962
1999	133,429	44,744	24,388
2000	136,048	46,107	25,753
2001	133,247	45,628	27,225
2002	129,518	44,203	26,604
2003	130,850	45,321	28,779
2004	129,710	46,159	27,501
2005	127,518	46,824	27,572
2006	127,537	48,610	30,231

Source: Vital statistics natality records covering all live births in Michigan from January 1, 1993 to December 31, 2006.

Table 2 presents results from the regression analysis. We report the relative risk ratios from multinomial logit models for the categories of “one parent on birth certificate” (which refers to unmarried mothers without paternity acknowledgement) and paternity acknowledgement, with “both parents on birth certicate” (i.e., married parents) as the base category. Results for “one parent on birth certificate” as the base category are available in Table S1 in the Supporting Information.

**Table 2:** Determinants of Paternity Acknowledgement at Childbirth, Michigan Births, 1993–2006.

	All Covariates				Child Gender Only			
	Unm. No Pat.		Pat. Ack.		Unm. No Pat.		Pat. Ack.	
	RRR	z	RRR	z	RRR	z	RRR	z
Child is Male	1.016***	3.007	1.039***	8.461	0.976***	−6.025	1.010**	2.527
Born on Weekend	1.047***	7.393	1.023***	4.191				
Any Abnorm. Cond./Cong. Anom.	1.020**	2.127	0.942***	−7.113				
Birth Weight (kg)	0.762***	−66.110	0.829***	−50.707				
Mother's Age: 20–24	0.289***	−133.151	0.322***	−130.644				
Mother's Age: 25–34	0.083***	−248.653	0.095***	−257.608				
Mother's Age: 35–44	0.066***	−195.526	0.073***	−213.214				
Mother's Age: 45+	0.055***	−24.554	0.054***	−28.146				
Mother's Ed: HS Degree	0.406***	−129.671	0.616***	−73.128				
Mother's Ed: Some College	0.203***	−188.241	0.384***	−127.531				
Mother's Ed: College+	0.046***	−220.659	0.098***	−220.848				
Mother's Ed: Missing	0.432***	−43.239	0.463***	−42.179				
Mother is Black	16.763***	444.230	4.788***	246.123				
Mother is Hispanic	1.068***	5.424	1.205***	18.605				
Mother is American Indian	2.373***	26.884	2.331***	30.807				
Mother is Other Race	0.644***	−21.951	0.502***	−41.615				
Second Parity	0.678***	−58.829	0.621***	−86.205				
Third Parity	0.787***	−29.274	0.614***	−68.323				
Fourth 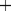 Parity	1.018*	1.916	0.641***	−50.969				
Birth Year	0.985***	−22.767	1.076***	129.730				
N	1,859,473		1,859,473		1,859,473		1,859,473	

Notes: Relative risk ratios (RRR) from multinomial logit models are reported. Outcome base 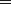 Both Parents on Birth Certificate (65.3% of all births). Outcomes: “Unm. No Pat” 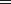 Unmarried, No Paternity Acknowledgement (16.5% of all births); “Pat. Ack.” 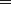 Paternity Acknowledgement (18.2% of all births). The sample of analysis is the universe of births in Michigan over 1993–2006. Standard errors are robust to heteroskedasticity. “Mother is Other Race” includes unknown. Omitted categories: mother's age 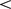 20; mother's education 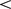 HS; mother's race/ethnicity is white; first parity.

Significance levels: *p 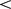 0.10 **p 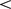 0.05 ***p 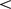 0.01.

The results show that births with paternity acknowledged have worse outcomes along various health and socio-economic dimensions relative to births to married parents, but better outcomes relative to births to unmarried parents without paternity acknowledgement. Relative to mothers with less than a high school education, mothers with a high school degree are 0.62 times as likely to have paternity acknowledged and only 0.41 times as likely to be unmarried with no paternity acknowledged. Mothers with a college degree or more are 0.10 times as likely to have paternity acknowledged and only 0.05 times as likely to be unmarried without paternity acknowledgement. These results imply that mothers with the highest education levels are married, mothers with intermediate education levels have paternity acknowledged, while mothers with the lowest education levels are unmarried without paternity acknowledged. A similar socio-economic gradient holds for maternal age: relative to mothers aged less than 20 years, mothers aged 20–24, 25–34, 35–44, and 45 or more years are 0.32 (0.29), 0.10 (0.08), 0.07 (0.07), and 0.05 (0.05) times as likely to have paternity acknowledged (be unmarried without paternity acknowledgement), respectively.

Relative to mothers who are non-Hispanic white, mothers who are black are 4.79 times more likely to have paternity acknowledged and 16.76 times more likely to be unmarried without paternity acknowledgement, while mothers who are Hispanic are 1.20 times more likely to have paternity acknowledged and 1.07 times more likely to be unmarried without paternity acknowledgement. Birth weight is also correlated with parental relationship status: relative to having both parents on the birth certificate, the relative risk ratios for paternity acknowledgement and unmarried without paternity acknowledgement are 0.83 and 0.76 per kilogram, respectively. Interestingly, the pattern is somewhat different for congenital anomalies or abnormal conditions: children with any congenital anomalies or abnormal conditions are 0.94 times as likely to have paternity acknowledged and are 1.02 times more likely to have unmarried parents without paternity acknowledgement. Children born on weekends are 1.02 times more likely to have paternity acknowledged and 1.05 times more likely to have unmarried parents without paternity acknowledgement. Finally, consistent with the evidence in Figure 1, there is an increasing trend in paternity acknowledgement over time: the relative risk ratio on a linear trend in the birth year is 1.08. Table S2 in the Supporting Information presents results where we control for only one dimension of socio-economic status or health at a time. In general, the individual dimensions have a stronger relationship with paternity acknowledgement in the absence of the full set of controls in Table 2, which is to be expected as these dimensions are correlated with each other and may also relate to paternity acknowledgement.

The result for child gender deserves particular attention. Absent strategic sex selection, one may argue that child gender is stochastic and in this respect approximates variation that might come from a randomized trial. Our results suggest that male children are more likely to have paternity acknowledged than female children – the relative risk ratio is 1.04 and statistically significant at the 1 percent level. Note that, consistent with previous studies, there are roughly 5% more boys than girls in our sample. Thus, more male newborns have paternity acknowledgement than females for two distinct reasons: i) they are more numerous than newborn girls, and; ii) for a given number of males, paternity establishment is more likely than for females. Our focus is on the latter effect.

Interestingly, the statistically-adjusted analyses suggest that males are more likely to have paternity acknowledged relative to *both* other relationship outcomes (see also Table S1). This is because we find that, in contrast with previous work, males are actually less likely to have parents who are married. However, the qualitative relationship between child gender and marriage is not robust as it flips signs depending on whether we include other covariates or not. In contrast, the relationship between child gender and paternity acknowledgement is relatively stable across all specifications. The sensitivity of the association between child gender and marriage suggests that this relationship may not necessarily be causal; the investigation of this question is left for future research.

## Discussion

Previous research has shown that marriage enjoys a host of positive correlates. Paternity acknowledgement provides an intermediate option between marriage and no legal relationship between parents. Despite becoming quite common in the U.S., relatively little is known about which parents choose paternity or how paternity is related to birth outcomes.

Overall, we find that average health and socioeconomic status of paternity births fall somewhere between those for married versus unmarried absent paternity. In the absence of a controlled experiment that assigns dimensions of health and socioeconomic status randomly and observes whether paternity is established, we cannot infer these correlates exert a causal effect on the decision to establish paternity. For example, it could be that low birth weight is correlated with a tenuous relationship between parents (unobserved in our data), and this tenuous relationship changes the likelihood that paternity is acknowledged.

While not eliminated, the problem of confounding from omitted variables bias might be less pronounced insofar as child sex is concerned. Compared with these other predictors of paternity, child sex is relatively stochastic. For example, our covariates do a very poor job at explaining overall variation in child sex, which is consistent with previous studies. Interestingly, a male child is positively associated with paternity regardless of the comparison group. Relative to married parents, a male child is 1% more likely to have his parents acknowledge paternity (no control variables). With multivariate controls, this magnitude increases to 3.9%. Either magnitude appears too large to be fully accounted for by reverse causality or the endogeneity of child sex as hypothesized by Trivers-Willard [13] – previous research finds that marriage is only responsible for a 0.2% increase in the likelihood of a male birth. [14]. That said, the magnitude of the positive relationship between male and paternity is more sensitive to statistical control than we expected. As noted above, we do not find that marriages are disproportionately male. Given the consistent positive relationship between male child and paternity, future work should explore whether acknowledging paternity is another dimension along which son preference is manifest.

## Supporting Information

Table S1
**Determinants of Paternity Acknowledgement and Marriage at Childbirth, Michigan Births, 1993–2006.** Notes: Relative risk ratios (RRR) from multinomial logit models are reported. Outcome base 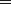 Unmarried, No Paternity Acknowledgement (16.5% of all births). Outcomes: “Pat. Ack.” 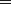 Paternity Acknowledgement (18.2% of all births); “Both Par.” 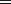 Both Parents on Birth Certificate (65.3% of all births). The sample of analysis is the universe of births in Michigan over 1993–2006. Standard errors are robust to heteroskedasticity. “Mother is Other Race” includes unknown. Omitted categories: mother's age 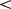 20; mother's education 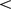 HS; mother's race/ethnicity is white; first parity. Significance levels: *p 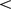 0.10 **p 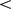 0.05 ***p 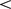 0.01.(PDF)

Table S2
**Determinants of Paternity Acknowledgement at Childbirth: By Separate Dimensions of Socio-Economic Status and Health.** Notes: Each column is from a separate regression. Relative risk ratios (RRR) from multinomial logit models are reported, with z-scores below. Outcome base 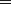 Both Parents on Birth Certificate (65.3% of all births). The sample of analysis is the universe of births in Michigan over 1993–2006. Standard errors are robust to heteroskedasticity. “Mother is Other Race” includes unknown. Omitted categories: mother's age 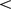 20; mother's education 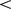 HS; mother's race/ethnicity is white; first parity. Significance levels: *p 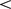 0.10 **p 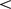 0.05 ***p 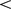 0.01.(PDF)
