# Supplementary material for: Paternity Acknowledgment in 2 Million Birth Records from Michigan
Source: PLoS One. 2013 Jul 22;8(7):e70042. doi: 10.1371/journal.pone.0070042 (PMC3718738; doi:10.1371/journal.pone.0070042)
Supplement: Table S1 — Determinants of Paternity Acknowledgement and Marriage at Childbirth, Michigan Births, 1993–2006. Notes: Relative risk ratios (RRR) from multinomial logit models are reported. Outcome base Unmarried, No Paternity Acknowledgement (16.5% of all births). Outcomes: “Pat. Ack.” Paternity Acknowledgement (18.2% of all births); “Both Par.” Both Parents on Birth Certificate (65.3% of all births). The sample of analysis is the universe of births in Michigan over 1993–2006. Standard errors are robust to heteroskedasticity. “Mother is Other Race” includes unknown. Omitted categories: mother's age 20; mother's education HS; mother's race/ethnicity is white; first parity. Significance levels: *p 0.10 **p 0.05 ***p 0.01. (PDF) [file pone.0070042.s001.pdf]

Table S 1. Determinants of Paternity Acknowledgement and Marriage at Childbirth, Michigan Births, 1993-2006

	**All Covariates**				**Child Gender Only**			
	**Pat. Ack.**		**Both Par.**		**Pat. Ack.**		**Both Par.**	
	**RRR**	**z**	**RRR**	**z**	**RRR**	**z**	**RRR**	**z**
Child is Male	1.023***	4.335	0.984***	-3.007	1.035***	6.855	1.025***	6.025
Born on Weekend	0.978***	-3.672	0.955***	-7.393				
Any Abnorm. Cond./Cong. Anom.	0.924***	-8.461	0.981**	-2.127				
Birth Weight (kg)	1.088***	20.719	1.312***	66.110				
Mother's Age: 20-24	1.113***	14.465	3.457***	133.151				
Mother's Age: 25-34	1.152***	16.116	12.100***	248.653				
Mother's Age: 35-44	1.112***	7.441	15.190***	195.526				
Mother's Age: 45+	0.986	-0.099	18.219***	24.554				
Mother's Ed: HS Degree	1.519***	63.926	2.466***	129.671				
Mother's Ed: Some College	1.890***	74.199	4.918***	188.241				
Mother's Ed: College+	2.105***	46.957	21.536***	220.659				
Mother's Ed: Missing	1.071***	3.372	2.315***	43.239				
Mother is Black	0.286***	-216.056	0.060***	-444.230				
Mother is Hispanic	1.128***	10.412	0.936***	-5.424				
Mother is American Indian	0.982	-0.607	0.421***	-26.884				
Mother is Other Race	0.779***	-10.884	1.553***	21.951				
Second Parity	0.916***	-13.159	1.474***	58.829				
Third Parity	0.780***	-29.061	1.270***	29.274				
Fourth or Higher Parity	0.630***	-46.738	0.982*	-1.916				
Birth Year	1.092***	132.970	1.015***	22.767				
N	1,859,473		1,859,473		1,859,473		1,859,473	

Notes: Relative risk ratios (RRR) from multinomial logit models are reported. Outcome base = Unmarried, No Paternity Acknowledgement (16.5% of all births). Outcomes: "Pat. Ack." = Paternity Acknowledgement (18.2% of all births); "Both Par." = Both Parents on Birth Certificate (65.3% of all births). The sample of analysis is the universe of births in Michigan over 1993-2006. Standard errors are robust to heteroskedasticity. "Mother is Other Race" includes unknown. Omitted categories: mother's age < 20; mother's education $<$ HS; mother's race/ethnicity is white; first parity.
Significance levels: *$p < 0.10$ **$p < 0.05$ ***$p < 0.01$
