# Supplementary material for: Paternity Acknowledgment in 2 Million Birth Records from Michigan
Source: PLoS One. 2013 Jul 22;8(7):e70042. doi: 10.1371/journal.pone.0070042 (PMC3718738; doi:10.1371/journal.pone.0070042)
Supplement: Table S2 — Determinants of Paternity Acknowledgement at Childbirth: By Separate Dimensions of Socio-Economic Status and Health. Notes: Each column is from a separate regression. Relative risk ratios (RRR) from multinomial logit models are reported, with z-scores below. Outcome base Both Parents on Birth Certificate (65.3% of all births). The sample of analysis is the universe of births in Michigan over 1993–2006. Standard errors are robust to heteroskedasticity. “Mother is Other Race” includes unknown. Omitted categories: mother's age 20; mother's education HS; mother's race/ethnicity is white; first parity. Significance levels: *p 0.10 **p 0.05 ***p 0.01. (PDF) [file pone.0070042.s002.pdf]

Table S 2. Determinants of Paternity Acknowledgement at Childbirth: By Separate Dimensions of Socio-Economic Status and Health

	Outcome: Paternity Acknowledged (18.2% of all births)								
	(1)	(2)	(3)	(4)	(5)	(6)	(7)	(8)	(9)
Child is Male	1.010**								
	2.527								
Born on Weekend		1.104***							
		21.306							
Any Abnorm. Cond./Cong. Anom.			0.956***						
			-6.362						
Birth Weight (kg)				0.682***					
				-125.364					
Mother's Age: 20-24					0.203***				
					-201.549				
Mother's Age: 25-34					0.038***				
					-417.396				
Mother's Age: 35-44					0.027***				
					-338.131				
Mother's Age: 45+					0.022***				
					-38.828				
Mother's Ed: HS Degree						0.370***			
						-180.441			
Mother's Ed: Some College						0.193***			
						-267.706			
Mother's Ed: College+						0.035***			
						-364.184			
Mother's Ed: Missing						0.254***			
						-84.116			
Mother is Black							4.890***		
							291.982		
Mother is Hispanic							2.335***		
							104.800		
Mother is American Indian							3.118***		
							49.841		
Mother is Other Race							0.431***		
							-56.576		
Second Parity								0.492***	
								-153.121	
Third Parity								0.454***	
								-132.397	
Fourth+ Parity								0.514***	
								-93.799	
Birth Year									1.052***
									106.372
N	1,859,473	1,859,473	1,859,473	1,859,473	1,859,473	1,859,473	1,859,473	1,859,473	1,859,473

Notes: Each column is from a separate regression. Relative risk ratios (RRR) from multinomial logit models are reported, with z-scores below. Outcome base = Both Parents on Birth Certificate (65.3% of all births). The sample of analysis is the universe of births in Michigan over 1993-2006. Standard errors are robust to heteroskedasticity. "Mother is Other Race" includes unknown. Omitted categories: mother's age < 20; mother's education $<$ HS; mother's race/ethnicity is white; first parity. Significance levels: *p < 0.10 **p < 0.05 ***p < 0.01
